# Supplementary material for: Choosing optimal trigger points for ex situ, in toto conservation of single population threatened species
Source: PLoS One. 2022 Apr 7;17(4):e0266244. doi: 10.1371/journal.pone.0266244 (PMC8989361; doi:10.1371/journal.pone.0266244)
Supplement: S1 File — (DOCX) [file pone.0266244.s001.docx]

**Supporting information for Brown *et al*. “Designing trigger points for *ex situ, in toto* conservation of single population threatened species”.**

Here, we provide the historical and current conservation context, the abundance timeseries, and the reference article, for each of the four case-studies analysed in the manuscript.

**DECLINE CASE STUDY 1: the North American wood turtle *Clemmys insculpta***

CONSERVATION CONTEXT

Garber & Burger (1995) provide a dataset describing the decline of a North American wood turtle population, in a protected wildlife reserve in Connecticut between 1982 and 1992. The population was distributed over two areas of this forest, with limited dispersal between them. Populations were stable until the park was opened to recreational use in 1982, when the dataset began. The population exhibited a decade of steady decline until its extinction in 1992. The size of the forest remained the same over this time, with no new roads being built and water and air quality remaining constant.

DECLINE DATASET

Table S1 shows a digitised version of Figure 1 from in Garber & Burger (1995). This data is shown in Figure 2 of the main text. We fit a linear decline model $N\left( t \right)=N\left( 0 \right)\left( 1+rt \right)$to the early phase of the decline, between 1982 and 1987 (inclusive). From this initial data, the best estimate of the decline rate is $r=-0.0965$, with 95% confidence bounds $[-0.1357, -0.05734]$.

***Table S1:*** *Abundance timeseries for the North American wood turtle (Garber & Burger, 1995, reproduced with permission)..*

| Survey year | 1982 | 1983 | 1984 | 1985 | 1986 | 1987 | 1988 | 1989 | 1990 | 1991 | 1992 |
| --- | --- | --- | --- | --- | --- | --- | --- | --- | --- | --- | --- |
| Population Estimate | 106 | 102 | 78 | 68 | 66 | 58 | 45 | 36 | 23 | 14 | 0 |

**DECLINE CASE STUDY 2: the vaquita *Phocoena sinus***

CONSERVATION CONTEXT

The vaquita was discovered in the late 1980s with the first surveys conducted in the 1990s (Rojas-Bracho & Jaramillo-Legoretta 2009). The biggest contributor to its population decline is bycatch mortality from gillnet fishing. Even a partial ban on gillnet fishing in 2015, and the establishment of a gillnet exclusion zone in 2017 has not arrested its decline (Jaramillo-Legorreta *et al.* 2019). The population is critically endangered with an abundance of fewer than 19 individuals (CIRVA 2019).

DECLINE DATASET

Table S2 shows timeseries data on the species decline, digitised from Figure 5 in Rojas-Bracho & Jaramillo-Legoretta (2009). This data is shown in Figure 2 of the main text. The population estimates for the Vaquita show a steady decline in population over the 21-year survey period. We fit a linear decline model $N\left( t \right)=N\left( 0 \right)\left( 1+rt \right)$to the early phase of the decline. From this data, the best estimate of the decline rate is $r=-0.041$, with 95% confidence bounds $[-0.039, -0.043]$.

***Table S2:*** *Abundance timeseries for the vaquita (Jaramillo-Legoretta, 2009, reproduced with permission).*

| Survey year | 1992 | 1993 | 1994 | 1995 | 1998 | 2000 | 2002 | 2003 | 2005 | 2008 | 2009 | 2013 | 2019 |
| --- | --- | --- | --- | --- | --- | --- | --- | --- | --- | --- | --- | --- | --- |
| Pop^n^ estimate | 700 | 684 | 665 | 634 | 533 | 469 | 400 | 370 | 309 | 230 | 216 | 131 | 19 |

**DECLINE CASE STUDY 3: the Christmas Island pipistrelle *Pipistrellus murrayi***

CONSERVATION CONTEXT

The Christmas Island Pipistrelle was common at the start of the 20^th^ century, and remained common until observations in 1984 showed that the population was rapidly declining. Unsure of whether the negative impacts were from invasive species or disease, the pipistrelle underwent normal monitoring until it was declared endangered in 2001. An intensive monitoring program was implemented from 2004 onwards in response. Captive breeding was not attempted until June 2009 when there was only 1 individual remaining (Martin *et al*. 2012).

DECLINE DATASET

Table S3 shows the population decline timeseries data for the pipistrelle, digitised from Figure 1 in Lunney *et al*. (2011). This data is shown in Figure 2 of the main text. A linear decline model $N\left( t \right)=N\left( 0 \right)\left( 1+rt \right)$ was fit to the data from the start of monitoring from 1994 to 2004. From this data, the best estimate of the decline rate is $r=-7.2119$ with large 95% confidence bounds $[-0.1552, -14.2685]$.

***Table S3:*** *Abundance timeseries for the Christmas Island pipistrelle (Lunney et al., 2011, reproduced with permission).*

| Survey year | 1994 | 1998 | 2002 | 2004 | 2005 | 2006 | 2007 | 2008 |
| --- | --- | --- | --- | --- | --- | --- | --- | --- |
| Population Estimate% | 100 | 71.15 | 42.31 | 27.88 | 20.67 | 13.46 | 6.246 | -0.97 |

**DECLINE CASE STUDY 4: the northern hairy-nosed wombat *Lasorhinus krefftii***

CONSERVATION CONTEXT

The only remaining wild northern hairy-nosed wombat *Lasorhinus krefftii* population resides in Epping Forest National Park, Central Queensland (a second population in Richard Underwood Reserve is probably too small and restricted to be considered independently viable). Abundance estimates have traditionally been based on burrow monitoring, and more recently using genetic analysis of hair samples. The species exhibited a fairly steady decline trajectory from the 1970s, reaching a nadir of 37 individuals in the early 1980s (Crossman, Johnson, & Horsup, 1994). Intensive management, including the removal of cattle from the area, alongside improved rainfall conditions, resulted in steady population growth throughout the 1990s.

DECLINE DATASET

We digitised two published abundance datasets, from 1980-1986 (Crossman, Johnson & Horsup 1994), and from 1987-1991 (Department of Environment and Energy 2016). This data is shown in Figure 2 of the main text, and in Table S4. The population estimates reveal the species’ abrupt declines in the late 1970s and early 1980s, followed by a recovery from approximately 1985 onward.

We fit a linear decline model $N\left( t \right)=N\left( 0 \right)\left( 1+rt \right)$to the early phase of the decline, starting from 1980. As there are two observations recorded per year, we record the second as occurring at the half-year point. From this initial data, the best estimate of the decline rate is $r=-16.82$, with 95% confidence bounds $[-25.06, -8.593]$.

***Table S4:*** *Abundance timeseries for the northern hairy-nosed wombat (Crossman, Johnson & Horsup, 1994, reproduced with permission).*

| Survey year | 1980 | 1980.5 | 1981 | 1981.5 | 1982 | 1982.5 | 1983 | 1983.5 | 1984 | |
| --- | --- | --- | --- | --- | --- | --- | --- | --- | --- | --- |
| Population Estimate | 111 | 113 | 80 | 79 | 49 | 69 | 36 | 45 | 46 | |
|  | | | | | | | | | | |
| Survey year | 1984.5 | 1985 | 1985.5 | 1986 | 1988 | 1988.5 | 1989 | 1989.5 | 1990 | 1991 |
| Population Estimate | 34 | 40 | 50 | 49 | 69 | 65 | 65 | 86 | 109 | 132 |

**REFERENCES CITED**

Crossman, D.G., Johnson, C.N., & Horsup, A.B. (1994). Trends in the population of the Northern Hairy-nosed Wombat *Lasiorhinus krefftii* in Epping Forest National Park, Central Queensland. *Pacific Conservation Biology* 1: 141-149.

Department of Environment and Energy (2016). Northern hairy-nosed wombat. *Department of Environment and Science.*

Garber, S.D., and Burger, J. (1995). A 20-yr Study Documenting the Relationship between Turtle Decline and Human Recreation.” *Ecological Applications* 5: 1151-1162.

Rojas-Bracho, L., & Jaramillo-Legoretta, A. M. (2009). Vaquita: Phocoena sinus. In *Encyclopedia of Marine Mammals* (pp. 1196-1200). Academic Press.

Jaramillo-Legorreta, A.M., Cardenas-Hinojosa, G., Nieto-Garcia, E., Rojas-Bracho, L., Thomas, L., Ver Hoef, J.M., & Tregenza, N. (2019). Decline towards extinction of Mexico's vaquita porpoise (Phocoena sinus). *Royal Society open science*, 6: 190598.

CIRVA (2019). Report of the Tenth Meeting of the *Comité Internacional para la Recuperación de la Vaquita* (CIRVA).

Lunney, D. Law, B. Schulz M. and Pennay, M. (2011). Turning the spotlight onto the Conservation of Australian bats and the extinction of the Christmas Island Pipistrelle. In: *The Biology and Conservation of Australasian Bats*. Eds: Law, B., Eby P., Lunney, D., Lumsde, L.

Martin, T.G, Nally, S., Burbidge, A.A., Arnall, S., Garnett, S.T., Hayward, M.W., Lumsden, L.F., Menkhorst, P., McDonald-Madden, E., & Possingham, H.P. (2012). Acting fast helps avoid extinction. *Conservation Letters* 5: 274-280
