# Supplementary figures and images for: Choosing optimal trigger points for ex situ, in toto conservation of single population threatened species
Source: PLoS One. 2022 Apr 7;17(4):e0266244. doi: 10.1371/journal.pone.0266244 (PMC8989361; doi:10.1371/journal.pone.0266244)

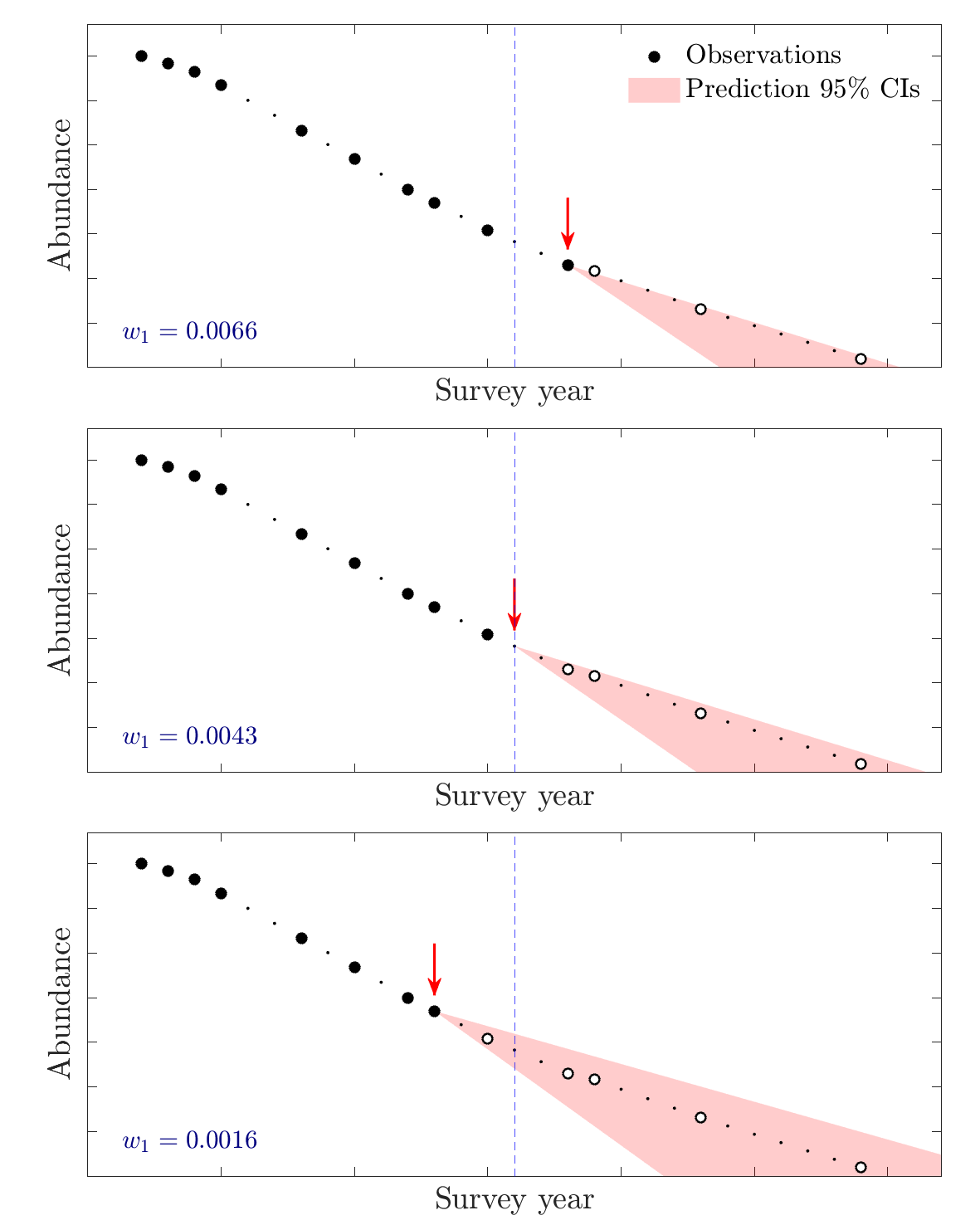

Supplement: S1 Fig — (TIF) [file pone.0266244.s002.tif]

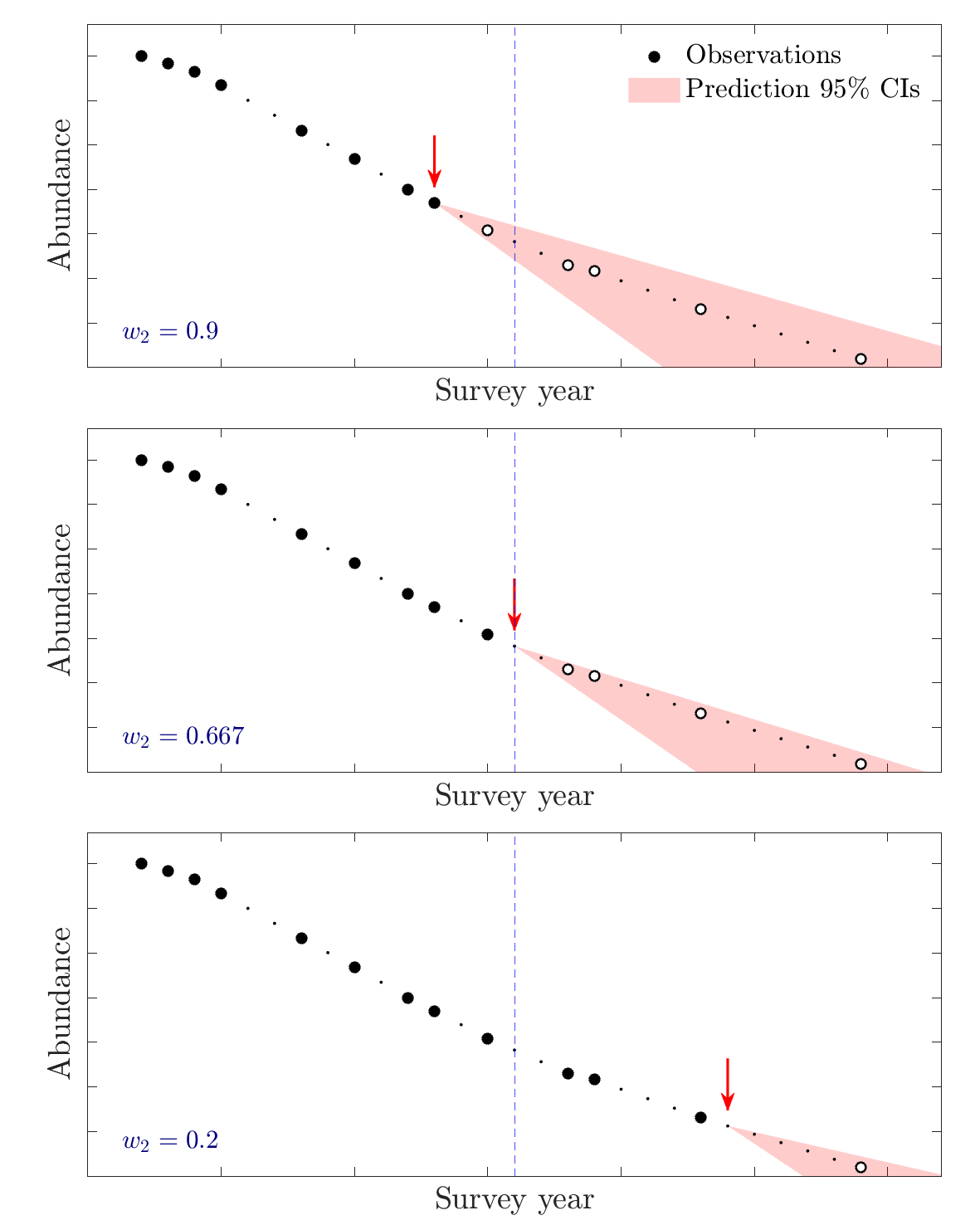

Supplement: S2 Fig — (TIF) [file pone.0266244.s003.tif]

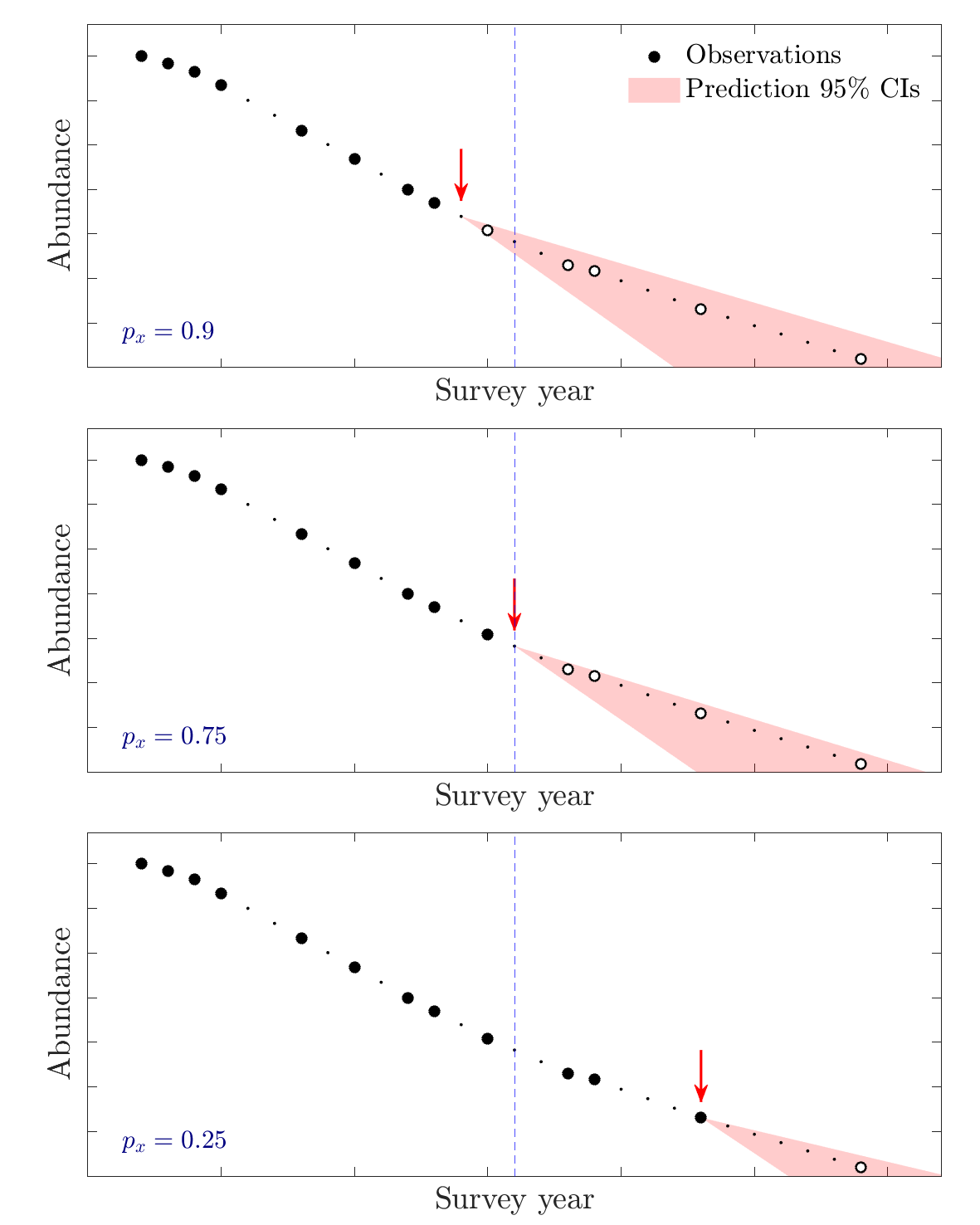

Supplement: S3 Fig — (TIF) [file pone.0266244.s004.tif]
