# Supplementary material for: Choosing optimal trigger points for ex situ, in toto conservation of single population threatened species
Source: PLoS One. 2022 Apr 7;17(4):e0266244. doi: 10.1371/journal.pone.0266244 (PMC8989361; doi:10.1371/journal.pone.0266244)
Supplement: Attachment — Submitted filename: Response_to_reviewers.docx [file pone.0266244.s005.docx]

**General comments**

We noted in your submission details that a portion of your manuscript may have been presented or published elsewhere.

Please clarify whether this [publication was peer-reviewed and formally published. If this work was previously peer-reviewed and published, in the cover letter please provide the reason that this work does not constitute dual publication and should be included in the current manuscript.

The data on species decline (i.e., the data shown in Figure 2) is taken from previously published papers. The sources of this data are all acknowledged, and all of the authors of these datasets have agreed to our use (see approvals in the cover letter).

It is thus not dual publication, since we are explicitly re-analysing data from a range of acknowledged sources for a different purpose.

**Reviewer 1**

This study introduces a decision support tool for deciding the optimal timing (trigger point) for transferring an entire population of a threatened species to captivity (ex situ, in toto). They use stochastic dynamic programming to determine trigger points. Every year the decision support tool assesses the expected population size in the following year based on the monitoring data (last population count and estimated rate of decline) as well as the probability that the next year’s attempt will be successful (based on the number of unsuccessful attempts of *in situ* actions). Whether the optimal timing for a trigger point is reached in any given year depends on the expected population size in the next year, the marginal value of next year's expected population size in terms of preventing extinction (w1), and how valuable it is to transfer the population *ex situ, in toto* compared to maintaining the current *in situ* management (w2). This seems to be a very interesting decision tool, and the paper is well written. My only concern is that the effects of w1 and w2 on finding trigger points are not evaluated, especially because they are based on stakeholders’ value system, which are highly subjective and could vary among different stakeholder groups.

Li 287: Can you be more specific and explain exactly what exactly you mean by “how stakeholders value in situ versus ex situ populations of different sizes”?

The referee makes an excellent point, and identifies an omission in our previous results. We have made several changes to address the issue. First, we have amended the manuscript to explain each parameter more clearly, and discuss how it influences the value function:

**Main text:** Smaller population sizes are more exposed to extinction, and have less genetic variation, even as they begin to recover. While managers always prefer larger populations, they are not twice as happy with a population that is twice as large. We use a parameter $w_{1}$ to control the rate at which larger populations deliver diminishing marginal benefits. Second, the value of a state should depend on whether the SPTS is *in situ*, or *ex situ, in toto* (with the former being preferable). We use a parameter $w_{2}$ to define their lower preference for the latter.

Second, we have undertaken additional analyses to measure how the parameters w1 and w2 affect optimal actions (i.e., the trigger points), as well as the parameter px. We describe these results in the main text, and provide figures illustrating their effects in the *Supporting Information*:

**Main text:** To evaluate the impact of varying our three arbitrary parameter choices (for $w_{1}$, $w_{2}$, and $p_{x}$), we repeated the optimisation process for values that were higher and lower than our previous selections.

**Main text**: The three parameters $w_{1}, w_{2}$, and $p_{x}$ each affect the timing of the optimal decision in predictable ways. For higher values of $w_{1}$, declining populations lose their value more slowly at first, allowing managers to wait longer before taking action (*Supporting Information Figure S1*). Higher values of $w_{2}$, which indicate a weaker preference for *in situ* populations, result in earlier *ex situ, in toto* action because managers are less reluctant to accept *ex situ* populations (*Supporting Information Figure S2*). Finally, higher values of $p_{x}$, which correspond to a higher likelihood of *ex situ, in toto* conservation success, encourage managers to attempt *ex situ* translocation earlier, since it carries less risk and is therefore a more attractive substitute for an in situ population (*Supporting Information Figure S3*).

Finally, we acknowledge the limitations of our value function:

**Main text:** Several of our parameter assumptions and prior belief distributions could be improved for particular threatened species. In particular, the two $w$ parameters in the value function depend significantly on the value stakeholders associate with *in situ* populations of different sizes, relative to the value placed on *ex situ* populations. Of course, where multiple stakeholders are involved, these values may not be shared by all participants.

Further, I wish the authors would have included the programming code to facilitate the implementation of this tool by population managers.

Agreed. We have now created a GitHub repository that contains Matlab code that can generates the primary set of results for our four case-studies. It also includes the four population abundance timeseries. We cite this repository in our article (line 251).

<https://github.com/MikeBode/SinglePopulationThreatenedSpecies>

Also not all the relevant literature has been cited, e.g. “Tenhumberg, B., Tyre, A. J., Shea, K., & Possingham, H. P. (2004). Linking wild and captive populations to maximize species persistence: optimal translocation strategies. Conservation Biology, 18(5), 1304-1314.”

Thanks for mentioning this citation – both because the analysis uses SDP, and because it applies the method to translocation between captive and wild populations. One of their solutions, in fact, involves the complete translocation of species from the wild to captivity (i.e., *ex situ, in toto*).

The papers are not completely overlapping, however. The most important difference is that the authors assume complete knowledge about the dynamics of both the captive and wild population dynamics – learning and uncertainty are not considered (note that stochasticity is addressed in this paper, but without any uncertainty about the underlying rates). These two issues are central to our work. We now highlight this previous research and results in the text.

**Main text:** While there are urgent calls for managers to set explicit trigger-points for drastic action when threatened species are in decline [13,14], there is limited theoretical guidance available to support this decision for SPTS. Previous optimisation analyses of translocations between captive and wild populations offer useful insights and decision-support, but they primarily consider translocations between wild and captive populations to be continual and/or reversible [17,18]. Tenhumberg *et al.* [17] find that *ex situ, in toto* emerges as an optimal solution to the question of captive breeding, but their analyses assume that the dynamics of both the captive and wild populations are known with certainty (i.e., that there is no parameter uncertainty), and again assume that animals can be shuttled continually between captive and wild populations. In contrast, the decision to initiate *ex situ, in toto* actions is fundamentally high-stakes, and high-uncertainty. It is critical that managers make the correct choice, but the irreversible and uncertain nature of the action makes it a very difficult decision.

Li 33: Replace “simple misfortunes” with “demographic stochasticity?

We have amended the statement to refer to stochasticity in general, rather than demographic stochasticity in particular, since other forms of stochasticity (e.g., environmental) can drive a population to extinction:

**Main text:** Natural population dynamics can push populations to low abundance, where they become vulnerable to Allee effects, genetic bottlenecks, or stochastic fluctuations.

Li 38: Infrastructure can't introduce new species. Better to write something like “Recently the development of hydroelectric infrastructure has been approved. This change may increase the chances of highly competitive species from adjacent catchment migrating into the creek.”

We have reworded the sentence to more accurately reflect the ecological process being described:

**Main text, line 37:** Australia’s stocky galaxias *Galaxias tantangara* (a listed species) are only found in the headwaters of Tantangara creek, and the approved development of hydroelectric infrastructure is may increase the chances of highly competitive species migrating into the creek from adjacent catchments, endangering *G. tantangara* [9].

Li 44: Replace “this habitat” with “its natural habitat”

We have amended the line accordingly.

Li 51: Replace “it is drastic step” with “it is a drastic step”; Li 54: Replace “was” with “were”

We have resolved the grammatical errors as suggested by the reviewer.

Li 78: I don’t think “psychological factors” is what you mean here.

We have removed the phrase.

Li 107: For consistency use numbers for citations

We have changed these citations to numbers.

The inset in Figure 1 is confusing. Wouldn’t the probability of in situ success decrease with the number of failed attempts n_u, in which case the x-axis should display n_u.

The inset figure is a plot of a belief distribution (the y-axis is a probability density), as a function of the probability of *in situ* success (the x-axis). We have amended the labels on the inset plot, and the caption text, to clarify this.

***Figure 1****: Schematic of an* ex situ, in toto *decision for a hypothetical single population threatened species (SPTS). The SPTS abundance has been observed to decline at a linear rate for 8 years; extrapolating, the population will likely drop to extinction within 4 – 10 years. The bars show the probability distribution for the extinction year, based on the range of linear declines shown in red. Conservation actions have been underway for 3 years without success. The inset plot shows the posterior belief distribution in the annual probability of* in situ *success, after 3 failures of in situ actions have occurred.*

Li 146: How exactly do you decide whether the in situ action has been successful? An increase in r, an increase in population size or no further decline in population size, …?

This is a good question, and a large assumption of our method. We make the assumption clear at the point we make it:

**Main text:** Moreover, we assume that if in situ actions successfully halt or reverse the decline, the managers will become aware of this fact within a year (i.e., one timestep).

And then we return to the point in the discussion:

**Main text:** Our choice of optimisation tools imposed limitations on our methods, which may limit its application, or reduce its optimality. For example, we assumed that, once the *in situ* actions are successful, managers become immediately aware of the fact. However, declining timeseries can include interregnums of apparent increase (e.g., the northern hairy-nosed wombat during 1983–1984; Figure 2). As a consequence, the positive proof of *in situ* success may need to come from very strong increases, or alternative evidence (e.g., substantial new recruitment into the population, or the successful eradication of a primary threat).

Li 178: Change “final” for “last”?

We have amended the text as per the reviewer’s suggestion:

Equation 3: you did not define r.

We have included a definition of the parameter $r$ after equation 3:

Li 200: Change the word “armed”

Li 202: Change “can calculated” to “can be calculated”.

We have changed the text as per the reviewer’s suggestions:

Li 207: Which programming language did you use and it would be nice if you would make the code available to the reader.

As mentioned above, we have made the code available in a Github repository, which we will link in the published manuscript. We have also added the following sentence to indicate which programming language we used.

**Main text:** We implement this procedure using MATLAB (ver. R2019b).

Li 223-224: “…we assume that managers have a relatively high satisfaction with the initial conditions of the in situ population, …” What exactly do you mean by this? Do managers believe the species can be successfully bred in captivity?

This is an arbitrary choice, and we now make this clear in the text:

**Main text:** For each case-study, we choose parameters for the value function that are arbitrary but plausible … we assume that managers were relatively satisfied with the abundance of the in situ population before the onset of decline. Specifically, we assume that they were 95% satisfied, and therefore that $w_{1}=\ln(20)/N(t_{o})$.

Li 248: Change “, but they only in …” to “, but only in …”

We have amended the grammatical error:

Li 263: Omit “in the past”? You claim “that a key driver of this inaction is a lack of effective planning.” However, limited funding and prioritizing to save other species might be more important reasons for inaction.

Agreed – we have omitted the phrase.

Li 285-286: You write “prior belief distributions could be improved with more information about the threatened species. “ Exploring the effect of this on determining trigger points would be very informative.

We agree that this would be an interesting extension, but do not have evidence to support an informative prior for any of our species. We hope to include this factor in a project we are now working on with the translocation of a marsupial species.

**Reviewer 2**

The authors proposed a theoretical framework to compute the optimal time step, at which managers should shift from in situ to ex situ, in toto conservation actions. The article is well written and the method is nice and clear. However the framework is based on several simplifications that might be too strong for this work to be used in practice. But it definitely provides an interesting tool to develop arguments and discuss this difficult problem of managing SPTS.

Thank you for reviewing our paper and providing feedback. We recognise the limitations of our methodology in practical application and have amended the manuscript (as mentioned in our response to the first reviewer’s comments) to elaborate where the parametrisation might be best improved.

The objective of this work is clearly stated in the introduction, which is noticeable. I would also like to see a paragraph where you discuss the differences between a SPTS conservation problem and other conservation problems. Could we use model from one problem to the other? Is there any major mathematical differences? Could your work apply to any conservation problem? You might add this paragraph in the discussion section if more appropriate.

E*x situ, in toto* conservation of a SPTS is an optimal stopping problem, characterised by irreversibility. In this sense it differs from other optimal learning decisions, which fit into the “explore-exploit” framework of adaptive management or reinforcement learning.

Rather than claim that our method – which we developed specifically for this SPTS application – should be applied to different problems, we discuss this difference in the discussion, drawing comparisons with other conservation methods.

**Main text:** The *ex situ, in toto* management of a threatened species is an irreversible decision, and must therefore be approached with caution by conservation managers. This is particularly true when there is uncertainty about both the likely success of both *in situ* and *ex situ* actions. The optimisation methods most suitable for the SPTS problem – and for many trigger-point problems – are therefore “optimal stopping” methods [26], which delay a critical action while more information is gathered. Optimal stopping problems differ substantially from adaptive management methods, where actions and observations occur continuously in a reinforcement learning loop. The latter are more common in conservation biology [17,23,24].

You should discuss the influence of the time step. Why a yearly time-step? Is it always appropriate, can it be changed? You are using a Markovian framework. Is the result of a management action can only be expected in the next year? Can it be in the following years as well? Is the way you model E[q(nu)] have any influence on the Markovian assumption?

This is a good point, and we have added text to the discussion to highlight this particular limitation with both our Markovian formulation, and therefore also our SDP methods:

**Main text:** Our choice of optimisation tools imposed limitations on our methods, which may limit its application, or reduce its optimality. For example, we assumed that, once the in situ actions are successful, managers become immediately aware of the fact. However, declining timeseries can include interregnums of apparent increase (e.g., the northern hairy-nosed wombat during 1983–1984; Figure 2). As a consequence, the positive proof of in situ success may need to come from very strong increases, or alternative evidence (e.g., substantial new recruitment into the population, or the successful eradication of a primary threat). If managers had to wait for several years of increase before they could conclude that in situ actions had been successful, or if it was known that these actions would take some time to be effective (e.g., if the management action was habitat restoration, which has a substantial time-lag), then the problem would cease to be Markovian, and a technique like SDP would become unsuitable.

L. 132, explain clearly what is success/failure. If possible, and needed, give some possible extension of your framework here, can it supports different definition of success/failure? Discuss the difficulty of defining success/failure in practice.

As mentioned above in our response to reviewer 1, we have amended the text to include an explicit definition of success in the text (and the difficulty of doing this). We make the assumption clear at the point we make it:

**Main text:** Moreover, we assume that if in situ actions successfully halt or reverse the decline, the managers will become aware of this fact within a year (i.e., one timestep).

And then we return to the point in the discussion:

**Main text:** Our choice of optimisation tools imposed limitations on our methods, which may limit its application, or reduce its optimality. For example, we assumed that, once the *in situ* actions are successful, managers become immediately aware of the fact. However, declining timeseries can include interregnums of apparent increase (e.g., the northern hairy-nosed wombat during 1983–1984; Figure 2). As a consequence, the positive proof of *in situ* success may need to come from very strong increases, or alternative evidence (e.g., substantial new recruitment into the population, or the successful eradication of a primary threat).

L.135 - why a beta distribution, give brief insights.

We have included the following sentence to explain the use of the beta distribution:

**Main text:** If in situ management actions have been unsuccessful for n_u years, then the probability that the next year’s attempt will be successful is the beta distribution $f(x;\alpha=1,\beta=n_{u}+1)$, since the beta distribution is a conjugate distribution of the binomial distribution.

L. 159, state what are v and v. I guess it is union and intersection?

We have included a statement to clarify the notation used in equation 1:

**Main text:** Where $\vee$ denotes the union and $\wedge$ denotes the intersection.

L. 241. Explain why there is so much uncertainty for the wood turtle case, it might help the reader understand more deeply the framework.

We appreciate the suggestion and have taken the opportunity to elaborate on multiple different ways in which the datasets vary (including the reviewer’s suggestion):

**Main text:** We choose these datasets because they are published examples of declining populations with well-studied abundance timeseries, and because they exhibit a range of behaviours and final outcomes, including extinction. The vaquita population, for example, has shown more than 25 years of ongoing decline, and currently numbers fewer than 50 individuals. During this period its abundance has not been observed to increase. In contrast, observations of northern hairy-nosed wombats have gone through periods of increase and decrease, even during longer-term declines. Some of the datasets are quite consistent in their declines (the vaquita and pipistrelle), while the others are much more variable (the turtle and wombat). The latter result in more uncertain predictions of future dynamics, and therefore less certainty about how much time is available to managers before extinction occurs. Two of the species eventually declined to extinction (the pipistrelle and turtle populations), while the wombat population recovered after a period of precipitous decline, after in situ management actions and environmental changes saved the species.

Finally, I’m surprised there is not a more general discussion on the implication of this work. For example, I’d be curious to know if we can derive a simple decision rule that gives the maximal number of years one can wait before switching to ex-situ, given an initial abundance, rate of decline and px. I guess computing the optimal strategy is not too costly on a computer, such that the optimal strategy can be computed a large number of time on simulated data with various parameters, and then try to derive some generic decision rules, for example using decision tree. The yearly decline rate can be drawn from some probability distribution with fixed variance and mean.

We now explore the effects of the different parameters on the optimal decisions (see additions to the *Supporting Information*, and our comments to reviewer 1), however, we have not had much success in distilling the results of our analysis down to a simpler rule-of-thumb. As the reviewer points out, the calculations required to apply the model are modest (by modern numerical standards), and the computer code that implements them is simple. We hope that – following the suggestion of the first reviewer – by hosting the computer code we can encourage people to use the full model (we are currently translating it into R).
